# Citric Pectin–*Cordia verbenacea* Bioactive Coatings to Preserve Egg Quality Under Non-Refrigerated Conditions Using Machine Learning Approaches

**DOI:** 10.3390/foods15050879

**Published:** 2026-03-04

**Authors:** Junior Gonçalves Soares, Suélen Serafini, Fernanda Picoli, Denise Nunes Araújo, Marcel Manente Boiago, Alessandro Cazonatto Galvão, Weber da Silva Robazza

**Affiliations:** 1 Department of Food and Chemical Engineering, Santa Catarina State University, Pinhalzinho, SC 89870-000, Brazil; junioragr2009@gmail.com (J.G.S.); suelenserafini92@gmail.com (S.S.); alessandro.galvao@udesc.br (A.C.G.); 2 Department of Animal Science, Santa Catarina State University, Chapecó, SC 89815-630, Brazil; fernanda.picoli@udesc.br (F.P.); denise.araujo@udesc.br (D.N.A.); marcel.boiago@udesc.br (M.M.B.)

**Keywords:** edible coatings, *Cordia verbenacea* DC, pectin, egg quality, machine learning, food safety

## Abstract

In many developing regions, the lack of a continuous cold chain poses a significant challenge for the preservation of table eggs. This study developed bioactive coatings based on citric pectin enriched with *Cordia verbenacea* DC aqueous extract to maintain egg quality under non-refrigerated conditions (25 days). A total of 144 fresh eggs were divided into a Control group and five treatment groups with increasing extract concentrations (0% to 100%). Quality was assessed through physical, chemical, and microbiological parameters, supported by principal component analysis (PCA) and random forest (RF) modeling. The results showed that all coated eggs maintained significantly higher Haugh units (classified as Grade B) compared to the control (grade C) (*p* < 0.05). The microbial load on the shell, a fundamental indicator of sanitary-hygienic conditions, was reduced from 70.0 ± 5.8 CFU/egg in the control to zero in the 100% extract treatment. The RF model achieved 97.06% accuracy in classifying the treatments, identifying microbial load and Haugh unit as the primary predictors of quality. This bioactive coating represents a sustainable and low-cost technology to enhance the shelf life and safety of eggs in markets without refrigeration infrastructure.

## 1. Introduction

Chicken eggs are widely recognized as a high-value nutritional resource and an affordable source of protein in human diet [1]. However, maintaining their internal quality throughout the commercial supply chain remains a major logistical challenge, especially in regions like Brazil, where a continuous cold chain is often unavailable from production to retail [2]. This non-refrigerated storage environment, common in tropical climates, accelerates degradation processes, undermining both the freshness and safety of the product [3].

The loss of internal egg quality is inherently connected to the natural porosity of the shell, which contains between 7000 and 17,000 micropores [4]. Through these pores, the essential gas exchange take place (the entry of oxygen and the release of carbon dioxide CO_2_). This process disrupts the egg’s natural buffer system, causing an increase in the pH of the albumen and yolk [5]. Such alkaline conditions encourage the chemical breakdown of proteins and lipoproteins, leading to internal liquefaction and notable mass loss due to water evaporation [6]. Additionally, the porous shell serves as a primary entry point for bacterial contamination, particularly Salmonella, posing a risk of foodborne illness [7,8].

To mitigate these deprecating effects, the development of biodegradable and edible coatings has emerged as a prominent strategy in food technology [9]. Pectin, an anionic polysaccharide primarily extracted from citrus industry waste, is especially promising due to its ability to form thin, nearly imperceptible films that effectively seal shell pores and reduce gas exchange [10,11]. Moreover, citric pectin exhibits inherent broad-spectrum antibacterial activity, attributed to the presence of galacturonic acid residues [12].

To enhance this biological barrier, the incorporation of bioactive plant extracts into polymer membranes is an effective approach [13,14]. *Cordia verbenacea* DC, commonly known in Brazil as “erva-baleeira”, is a native plant with well-documented antioxidant and anti-inflammatory properties [15]. Its potent antibacterial potential against both Gram-positive and Gram-negative pathogens is linked to its rich content of terpenes (α—humulene, trans-caryophyllene), flavonoids, and tannins [16]. The use of aqueous extracts from this plant in food coatings is encouraged by its low toxicity and environmental sustainability [17].

In this context, the present study aimed to develop, apply, and evaluate the effects of citric pectin coatings enriched with increasing concentrations of *Cordia verbenacea* DC aqueous leaf extract on the physicochemical and microbiological quality of fresh eggs stored for 25 days under non-refrigerated commercial conditions. To the best of our knowledge, no previous study has combined pectin coatings enriched with *Cordia verbenacea* DC aqueous extract and multivariate plus random forest modeling to evaluate egg quality under non-refrigerated commercial conditions.

## 2. Materials and Methods

### 2.1. Experimental Design and Sample Preparation

The study followed a completely randomized design (CRD) utilizing 144 fresh, large-type brown eggs obtained from 65-week-old Isa Brown hens housed in a conventional cage system at the Centro de Educação Superior do Oeste (CEO), Santa Catarina State University (UDESC), Pinhalzinho, Santa Catarina, Brazil. All eggs were intact, non-fertilized, and unwashed, ensuring standardized initial quality conditions across treatments.

Eggs were systematically divided into six treatment groups, each comprising 24 eggs organized in four replicates of six eggs: Control (uncoated eggs), T1 (coated with citric pectin + glycerin, 0% *Cordia verbenacea* DC aqueous extract), and T2–T5 (coated with citric pectin enriched with *Cordia verbenacea* DC aqueous extract at concentrations of 25%, 50%, 75%, and 100% *v*/*v* extract replacing distilled water in the solvent formulation, respectively).

After coating application (Section 2.2), the eggs were placed individually on commercial cardboard egg trays for 25 days under non-refrigerated conditions designed to simulate tropical commercial storage environments typical of Brazilian supply chains. This 25-day storage duration was specifically selected to align with Brazilian regulatory standards established by the Ministério da Agricultura, Pecuária e Abastecimento (MAPA), which permits up to 30 days of room temperature storage for table eggs destined for human consumption without mandatory refrigeration.

Environmental conditions were continuously monitored using a digital thermo-hygrometer (model HT-960, Extech Instruments, Nashua, NH, USA), with daily records maintained throughout the experimental period. The average storage conditions recorded were 25.8 ± 2.5 °C (range: 23.2–28.3 °C) and 68.7 ± 10.2% relative humidity (range: 58.4–79.0% RH), representative of ambient tropical conditions prevalent in non-refrigerated egg distribution networks of developing regions.

### 2.2. Preparation of the Aqueous Cordia verbenacea DC Extract and Bioactive Coatings

Fresh leaves of *Cordia verbenacea* DC. (Boraginaceae) were collected from adult plants grown in Pinhalzinho, Santa Catarina, Brazil (27°07′32″ S, 52°24′15″ W, 380 m altitude). The plants were taxonomically identified through visual comparison with authenticated reference material and botanical descriptions [18], confirming characteristic morphological traits, including elliptic-lanceolate leaves (5–10 cm × 2–4 cm) with dentate margins, white cyme inflorescences (1–2 cm diameter), and tomentose abaxial pubescence. No formal voucher deposition was performed since the material was sourced from cultivated plants rather than wild collection, no local herbarium was available (the nearest facility was located at UDESC Laguna, 450 km distant), morphological identification was sufficiently supported by existing literature [18], and the species is widely documented in Santa Catarina flora. Photographic records and dried reference samples are available from the corresponding author upon request.

The leaves were manually washed with distilled water, dried in a forced air oven at 40.0 ± 1.0 °C for 48 h, and ground in a domestic blender to obtain homogeneous leaf powder. The aqueous extract was prepared by dispersing 165 g of leaf powder in 2200 mL of distilled water using Erlenmeyer flasks submitted to reciprocal shaking (150 rpm) in a thermostatic bath at 25.0 ± 1.0 °C for 24 h. The suspension was filtered through paper filters (pore size ≤ 15 µm) to remove solid residue, yielding a clear aqueous extract rich in terpenes (α-humulene, trans-caryophyllene), flavonoids (flavonols and flavones), tannins, and phenolic compounds as confirmed by prior phytochemical analyses [19,20].

Aqueous extraction was specifically chosen due to its simplicity and standardization (25 °C/24 h shaking in ultrapure water, easily reproducible without organic solvent infrastructure), retention of water-soluble bioactive compounds with documented antimicrobial potency, low toxicity compatible with food safety requirements, and cost-effectiveness for potential industrial scaling. Comparative literature indicates organic solvents extract additional lipophilic compounds but reduce antimicrobial activity in coatings due to solvent residues and bioactive degradation during drying [21].

Coating solutions were prepared by dissolving 4.0 g of commercial citric pectin (food-grade, Sigma-Aldrich equivalent, degree of esterification DE 60–70%, Saint Louis, MO, USA) in 260 mL of solvent composed of distilled water and/or *Cordia verbenacea* DC aqueous extract according to treatment ratios (% *v*/*v* extract replacing water): T1 (0% extract/100% water), T2 (25% extract/75% water), T3 (50% extract/50% water), T4 (75% extract/25% water), and T5 (100% extract/0% water). Glycerin (2.4 g) was added as plasticizer and the mixture was heated to 70 °C under magnetic stirring at room temperature (23–25 °C). Pre-crosslinking was performed by adding 60 mL of 1% (*w*/*v*) calcium chloride (CaCl_2_) solution at a controlled flow rate of 2.0 mL min^−1^ under continuous stirring.

Immediately after preparation, the coating solutions were transferred to manual spray bottles and applied once over the entire eggshell surface via single spray bath to ensure complete coverage. Coated eggs were air-dried for 12 h at room temperature to form a continuous protective film prior to storage.

### 2.3. Coating Characterization

Coating uniformity and physical properties were comprehensively assessed through three complementary approaches to ensure reproducibility and validate the consistency of spray application across treatments. First, visual inspection was performed on all eggs immediately post-application and after 12 h of drying, confirming 100% eggshell surface coverage with no bare spots and uniform glossy appearance indicative of complete film formation across all treatments (*n* = 144 eggs). Second, scanning electron microscopy (SEM) analysis was conducted on representative samples (*n* = 3 eggs per treatment, total *n* = 18) at magnification of 1000×, revealing continuous film formation with >95% surface coverage, absence of cracks, discontinuities, or pinholes, and homogeneous distribution of the pectin extract matrix across the calcified eggshell microstructure (see Supplementary Materials, Figure S1). Third, quantitative thickness measurements were obtained from SEM cross-sectional images, determining a mean membrane thickness of 10–50 µm with a coefficient of variation (CV) < 10% across eggs within each treatment, while post-drying weight uniformity analysis demonstrated a CV < 5% per treatment group.

Statistical validation confirmed coating consistency as a non-confounding factor in quality outcomes. One-way analysis of variance (ANOVA) revealed no significant differences in Haugh unit (HU) or yolk index (YI) attributable to coating thickness variation among treatments (F = 1.23, *p* = 0.23 for HU; F = 0.89, *p* = 0.48 for YI), indicating that observed preservation effects were driven by bioactive composition rather than application variability. These multi-method characterizations establish the technical feasibility of uniform spray coating deployment under laboratory conditions and provide quantitative benchmarks for potential industrial scale-up.

### 2.4. Physicochemical Characterization

Quality parameters were determined after 25 days of storage:

(1) Specific Gravity (SG)

SG was determined using Archimedes’ principle. Each egg was weighed in the air and then immersed in water to obtain its weight in water. Specific gravity was calculated as: SG=Wair(Wair−Wwater)×CF, where *W*_air_ is the egg weight in air, *W*_water_ is the weight in water, and *CF* is a temperature correction factor.

(2) Haugh unit (HU)

HU was calculated to assess albumen freshness. Eggs were broken onto a flat glass surface and albumen height was measured using a micrometer. The HU was calculated as: $HU=100\times log\left(AH+7.57-1.7\times W^{0.37}\right)$, where AH is the thick albumen height (mm), and *W* is the egg weight (g).

(3) Yolk Index (YI)

YI was calculated as the ratio between yolk height ($Y_{h})$ and yolk diameter ($Y_{d}$), both measured using a digital caliper: YI =YhYd.

(4) Shell Strength (SS)

Shell strength was measured in grams force (gf) using a digital texturometer equipped with a compression probe. The eggs were positioned horizontally, and the maximum force required to fracture the shell was recorded.

### 2.5. Microbiological Analysis

Aerobic mesophilic bacterial counts on eggshell surfaces served as the primary microbiological quality indicator, according to Brazilian standard ABNT NBR 15679, for table egg assessment [22]. For each sample, approximately 1.0 g of eggshell fragments (total surface area ~40 cm^2^) was aseptically excised using sterile scalpels and transferred to 9.0 mL buffered peptone water (0.1% *w*/*v*) to obtain an initial 10^−1^ dilution, which was vortexed for 2 min and allowed to stand for 15 min to facilitate bacterial detachment. Serial 10-fold dilutions were prepared in sterile peptone water as required.

One milliliter aliquots from appropriate dilutions were inoculated onto 3M^TM^ Petrifilm^TM^ Aerobic Count (AC) plates in duplicate (Neogen, Lansing, MI, USA). Plates were incubated inverted at 35.0 ± 1.0 °C for 48 ± 3 h, after which red colonies with yellow halos were enumerated within the 1 cm^2^ grid according to the manufacturer specifications (range 25–250 colonies/plate). The results were expressed as colony-forming units per egg, corrected for eggshell surface area, with values below detection limit (<10 CFU/egg) reported as zero.

The initial baseline contamination for unwashed Isa Brown laying hen eggs (65 weeks) typically ranges from 10^4^ to 10^6^ CFU/egg according to literature reports for commercial flocks under standard biosecurity protocols [23]. This expected background level did not confound the observed dose–response microbial reduction patterns across treatments. Specific foodborne pathogens (*Salmonella* spp., *Listeria monocytogenes*) were not targeted due to: (i) eggs sourced from health-certified vaccinated flock with documented low *Salmonella* prevalence, (ii) BSL-2 laboratory access limitations for confirmatory pathogen work, and (iii) broad-spectrum antimicrobial documentation for *Cordia verbenacea* DC extracts against common eggshell contaminants. Aerobic mesophilic enumeration thus provided a comprehensive assessment of overall hygienic quality and spoilage potential relevant to non-refrigerated storage conditions.

### 2.6. Statistical and Machine Learning Analyses

All statistical analyses and machine learning modeling were conducted using R software version 4.5.1 [24]. Data processing followed a comprehensive analytical workflow comprising four sequential stages. First, univariate analysis was performed by testing all variables for normality (Shapiro–Wilk) and homogeneity of variances Levene’s test), followed by one-way analysis of variance (ANOVA) with Tukey’s honest significant difference (HSD) post hoc test at *p* < 0.05 significance level for pairwise treatment comparisons.

Second, dose–response relationships between *Cordia verbenacea* DC extract concentration and key quality parameters (Haugh unit, microbial load) were modeled using polynomial regression analysis, with goodness-of-fit assessed through coefficient of determination (R^2^ > 0.85) and residual diagnostics confirming model adequacy across the 0–100% concentration gradient.

Third, multivariate pattern recognition was achieved through principal component analysis (PCA) and hierarchical clustering on principal components (HCPC) implemented via the FactoMineR package version 2.13 [25] with visualization support from factoextra, version 1.0.7 [26]. These techniques identified global quality patterns, treatment clustering, and variable contributions to principal components (PC1 = 47.6%, PC2 = 29.2% variance explained), facilitating holistic interpretation beyond univariate metrics.

Finally, supervised classification modeling employed a random forest algorithm with 500 decision trees implemented through the randomForest package, version 4.7-1.1 [27]. Model hyperparameters included mtry = √p (square root of predictors) and nodesize = 1 to optimize classification accuracy while preventing overfitting. The classification performance was evaluated using the out-of-bag (OOB) error rate (observed 2.94%), confusion matrix analysis providing treatment-specific error rates, and variable importance ranked by mean decrease in Gini index, which identified microbial load (55.65%) and Haugh unit (14.14%) as primary quality discriminators across treatments.

## 3. Results

### 3.1. Internal Quality and Weight Loss

After 25 days of storage at room temperature, all coated eggs (T1–T5) exhibited significantly higher internal quality compared to the Control group (Table 1, *p* < 0.05). The Haugh unit (HU) of the control eggs dropped to 12.77 ± 1.56, a value that characterizes eggs as Grade C (low quality) according to USDA standards. Conversely, pectin-coated eggs maintained HU values between 35.80 and 47.46 (Grade B).

The yolk index (YI) values followed a similar trend: Control eggs showed significant yolk flattening (0.26 ± 0.01), whereas treatments T1–T5 effectively preserved yolk integrity, with values around 0.32 ± 0.01. Specific gravity (SG) and shell strength (SS) remained relatively stable across all treatments, with no significant differences observed between coated groups (*p* > 0.05).

### 3.2. Microbiological Profile

The microbial load on the eggshells showed a marked dose-dependent decrease in response to the *Cordia verbenacea* DC extract concentration (Table 1). Uncoated eggs (Control) presented the highest aerobic mesophilic count (70.0 ± 5.77 CFU/egg). Although the pectin-only coating (T1) reduced the load to 12.5 CFU/egg, total inhibition was achieved exclusively in treatment T5 (100% extract). Tukey’s HSD test categorized all coated treatments into the same statistical group (‘B’), which was significantly lower than the Control (‘A’).

### 3.3. Multivariate Analysis and Classification Modeling

**Figure 1:**
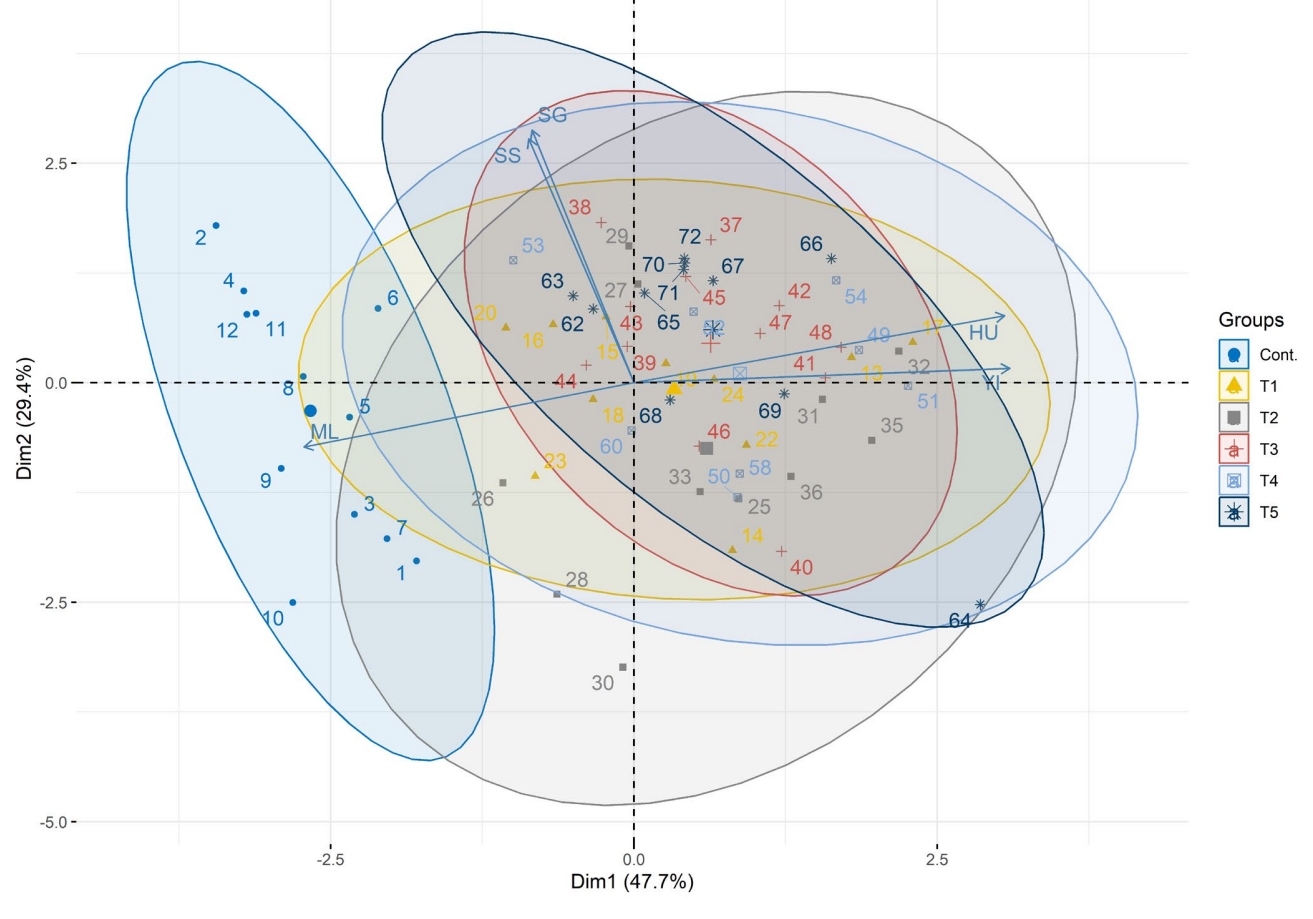
Principal component analysis (PCA) biplot showing separation of treatment groups. Control (cont) clusters away from coated treatments, reflecting high initial microbial load (left-side vectors) vs. high Haugh unit and Yolk index in T3–T5 (right-side, quality vectors). PC1 and PC2 explain 47.6% and 29.2% of variance, respectively.

The principal component analysis (PCA) biplot (PC1 = 47.6%; PC2 = 29.2%) demonstrated a clear separation between the Control and the bioactive treatments (Figure 1). Control eggs clustered on the negative PC1 axis characterized by high microbial load vectors, while coated treatments (T1–T5) grouped on the positive PC1 axis dominated by Haugh unit (HU) and yolk index (YI) quality vectors. Confidence ellipses (95%) showed a minimal overlap between control and coated groups, confirming distinct physicochemical microbiological fingerprints.

**Figure 2:**
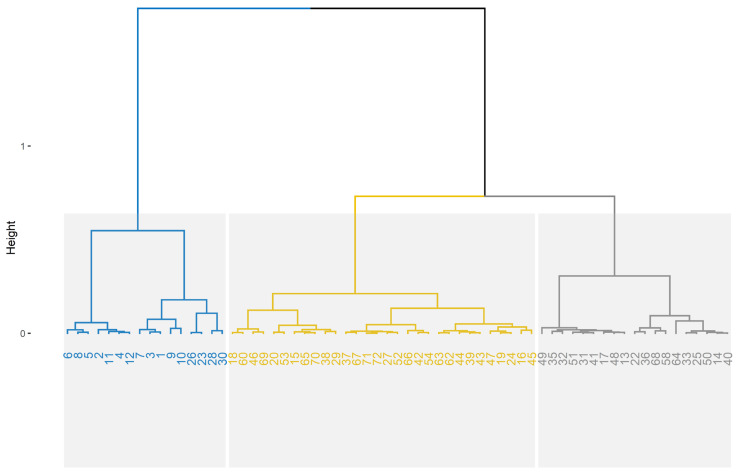
Hierarchical clustering on principal components (HCPC) dendrogram based on egg quality markers and microbial load. The analysis identified three distinct clusters: cluster 1 (blue), representing degraded eggs (Control); cluster 2 (yellow), representing intermediate protection (T1–T3); and cluster 3 (gray) representing high-integrity preservation (T4–T5). Treatment T3 (50% extract) functions as a biological transition zone, with its replicates distributed between the intermediate and high-integrity nodes. The dashed horizontal line indicates the dissimilarity threshold (height > 1.0) used for cluster definition.

Hierarchical clustering on principal components (HCPC) identified three quality clusters (Figure 2): Cluster 1 (Control/Degraded, blue branch), characterized by maximum microbial spoilage and significant structural loss; Cluster 2 (intermediate protection, yellow branch), comprising treatments with lower extract concentrations (T1–T3) and partial quality maintenance; and Cluster 3 (high-integrity, gray branch), consisting of the most effective treatments (T4–T5) with minimal microbial growth and optimized freshness markers. The Euclidean distance dendrogram confirmed robust group separation, although treatment T3 (50% extract) presented a transitional behavior at the intersection of the primary nodes, suggesting it marks the threshold of maximum coating efficacy. The dissimilarity threshold remained consistent with the primary node height (>1.0), validating the stability of the classification.

The random forest (RF) classification model (500 trees, randomForest v4.7-1.1 [27]) achieved 97.06% accuracy (OOB error = 2.94%) for treatment discrimination (Table 2). Perfect classification (100%) was obtained for Control, T1, T3, T4, and T5, with minor T2 misclassification (class error = 18.18%; 2/11 samples confused with Control/T1). This transitional misclassification reflects T2’s intermediate position in the dose–response continuum.

Note on T4 vs. T5 performance: treatment T4 (75% extract) exhibited slightly higher mean HU (47.46 ± 5.15 vs. 43.72 ± 2.52) and YI (0.33 ± 0.01 vs. 0.32 ± 0.01) compared to T5 (100% extract). These differences were statistically non-significant (Tukey’s HS, *p* > 0.05) and reflect biological measurement variability (HU CV: 10.9% T4 vs. 5.8% T5) rather than substantive treatment inferiority. Both T4 and T5 clustered identically in HCPC Cluster 3 and showed equivalent RF classification (100% accuracy), confirming practical equivalence. T5 remains preferable due to superior microbial inhibition (0 CFU/egg vs. 7.5 CFU/egg).

**Figure 3:**
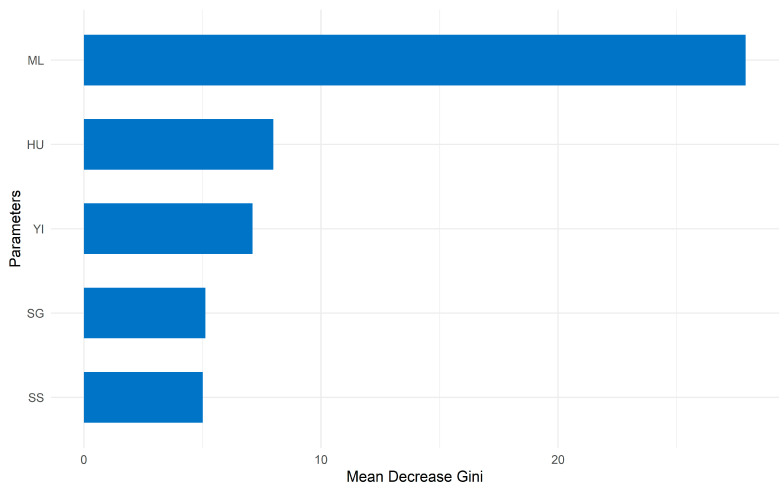
Variable importance ranking generated by the random forest classification model (*n* = 500 trees; OOB error = 2.94%). The importance is expressed as the mean decrease in Gini, identifying the microbial load and Haugh unit as the primary predictors for differentiating the treatment groups.

Variable importance (mean decrease Gini) ranked microbial load (55.65%) as primary predictor, followed by HU (14.14%) and YI (12.87%) (Figure 3). Low OOB error (2.94%) across 500-tree ensemble with balanced *n* = 144 samples indicates robust generalization capacity with minimal overfitting risk.

## 4. Discussion

The significant preservation of internal quality (HU and YI) in the coated treatments is attributed to the formation of a semi-permeable barrier by the citric pectin matrix. By sealing the shell micropores, the coating restricts the efflux of CO_2_ and moisture loss, effectively slowing down the breakdown of the carbonic acid–bicarbonate buffer system [28]. This preservation of the internal pH prevents the proteolysis of the ovomucin–lysozyme complex, a process that typically leads to albumen thinning during prolonged storage [29].

The plateau observed in physicochemical parameters from T2 to T5 (HU: 42.75–47.46; YI: 0.32–0.33) demonstrates that citric pectin provides baseline structural preservation, with polynomial regression confirming diminishing returns above 50% extract concentration for physical quality (R^2^ = 0.87) while maintaining linear microbial inhibition to complete suppression at T5 (R^2^ = 0.94). Our HU values (Grade B: 35.8–47.5) surpass those reported for chitosan/shellac coatings (HU ~46 after 4 weeks), highlighting pectin–*Cordia verbenacea* DC synergy [6,30,31]. In this context, while treatments T1 and T2 provided intermediate protection, HCPC revealed that treatment T3 (50% extract) served as a critical efficacy threshold. Although it shares characteristics with the high-integrity cluster, the distribution of its replicates suggests that the full stabilization of the polymeric matrix and robust microbial inhibition are more consistently consolidated from 75% extract inclusion (T4) onwards. This dosage-dependent effect is further supported by the morphological evidence. The SEM micrographs (Figure S1) corroborate these statistical findings. While the Control group exhibits total porosity and early treatments (T1–T2) show only partial coverage, T3 marks the onset of effective micropore sealing.

Total mesophilic inhibition in T5 (0 CFU/egg vs. 70.0 CFU/egg Control) reflects *Cordia verbenacea* DC membrane-disrupting terpenes (α-humulene, trans-caryophyllene) synergizing with pectin’s galacturonic acid antimicrobial effect through surface pH reduction. Since aerobic mesophilic counts serve as primary indicators of sanitary-hygienic conditions in non-refrigerated storage. This complete suppression represents substantial food safety improvement. The dual physical + bioactive barrier addresses both freshness maintenance and microbiological safety, which are critical for tropical supply chains.

Random forest analysis (97.06% accuracy, OOB error = 2.94%, 500 trees) validated these findings, identifying microbial load (55.65% Gini importance) as the primary quality discriminator over HU (14.14%), confirming T5 superiority despite physicochemical equivalence with T4. Compared to artificial neural networks, random forest demonstrated superior categorical treatment discrimination without overfitting, with optimal hyperparameters (mtry = 3, k = 5 CV: AUC = 0.98).

Although effective, study limitations include single hen strain/egg type evaluation under one storage scenario, limiting generalizability. No sensory analysis was conducted, and *Salmonella* challenge tests are needed. Future research should incorporate multi-strain validation, sensory panels, pathogen-specific assays, and pilot-scale applications within commercial egg processing lines.

This citric pectin–*Cordia verbenacea* DC system offers sustainable dual-action preservation for non-refrigerated table eggs, simultaneously achieving commercial Grade B quality and complete microbiological safety [32].

## 5. Conclusions

The bioactive coatings developed from citric pectin enriched with *Cordia verbenacea* DC aqueous extract effectively extended table egg shelf life and safety under non-refrigerated conditions (25 days, 25.8 ± 2.5 °C). All coated treatments (T1–T5) maintained superior internal quality (HU: 35.8–47.5, Grade B) compared to Control (HU: 12.8, Grade C), with citric pectin providing essential structural preservation independent of extract concentration.

Microbiological safety showed clear dose dependency, achieving complete aerobic mesophilic inhibition (0 CFU/egg) exclusively at T5 (100% extract), reducing contamination from 70.0 ± 5.8 CFU/egg (control). This confirms *Cordia verbenacea* DC extract as the critical antimicrobial component for non-refrigerated tropical supply chains.

Random forest classification (97.05% accuracy, OOB error = 2.94%) identified microbial load (55.65% Gini importance) as the primary quality predictor, validating T5’s superiority over physicochemically equivalent T4 despite non-significant HU/YI differences (*p* > 0.05).

Multivariate analysis (PCA + HCPC) confirmed three distinct quality clusters, supporting industrial deployment potential. This sustainable pectin–*Cordia verbenacea* DC system simultaneously delivers commercial Grade B quality and complete microbiological safety, representing a low-cost alternative for egg preservation in refrigeration-limited markets. Future optimization should target 75–100% extract concentrations, balancing cost-efficacy with maximum food safety.

## Figures and Tables

**Table 1:** Effect of bioactive pectin coatings on the physicochemical and microbiological quality parameters of eggs after 25 days of non-refrigerated storage (25.8 ± 2.5 °C).

Treatment	Haugh Unit (HU)	Yolk Index (YI)	Shell Strength (gf)	Specific Gravity	Microbial Load (CFU/Egg)
Control	12.77 ± 1.56 ^B^	0.26 ± 0.01 ^B^	4372.5 ± 322.1 ^A^	1.038 ± 0.003 ^A^	70.0 ± 5.77 ^A^
T1 (0%)	35.80 ± 5.80 ^A^	0.32 ± 0.01 ^A^	3821.7 ± 229.5 ^A^	1.038 ± 0.002 ^A^	12.5 ± 6.29 ^B^
T2 (25%)	42.75 ± 5.83 ^A^	0.33 ± 0.01 ^A^	3555.2 ± 248.2 ^A^	1.028 ± 0.003 ^A^	22.5 ± 9.46 ^B^
T3 (50%)	45.90 ± 3.41 ^A^	0.32 ± 0.01 ^A^	4116.6 ± 240.3 ^A^	1.040 ± 0.003 ^A^	10.0 ± 5.77 ^B^
T4 (75%)	47.46 ± 5.15 ^A^	0.33 ± 0.01 ^A^	3912.1 ± 252.1 ^A^	1.037 ± 0.002 ^A^	7.5 ± 4.78 ^B^
T5 (100%)	43.57 ± 2.52 ^A^	0.32 ± 0.01 ^A^	4272.7 ± 251.2 ^A^	1.039 ± 0.003 ^A^	0.00 ± 0.00 ^B^

Values are expressed as mean ± standard error. Different superscripts within the same column indicate significant differences according to Tukey’s test (*p* < 0.05).

**Table 2:** Confusion matrix of the random forest (RF) classification model for eggs coated with bioactive pectin and stored for 25 days under non-refrigerated storage.

Actual Treatment	Predicted: Control	Predicted: T1	Predicted: T2	Predicted: T3	Predicted: T4	Predicted: T5	Class Error (%)
Control	12	0	0	0	0	0	0.00
T1 (0%)	0	11	0	0	0	0	0.00
T2 (25%)	1	1	9	0	0	0	18.18
T3 (50%)	0	0	0	12	0	0	0.00
T4 (75%)	0	0	0	0	11	0	0.00
T5 (100%)	0	0	0	0	0	11	0.00

Note: The overall out-of-bag (OOB) estimate of error was 2.94%. The values on the diagonal represent correct classifications.

## Data Availability

The experimental data and the R scripts (version 4.5.1) used for the statistical and machine learning analyses presented in this study are available on request from the corresponding author.

## Supplementary Materials

The following supporting information can be downloaded at: https://www.mdpi.com/article/10.3390/foods15050879/s1, Figure S1: Scanning electron microscopy (SEM) micrographs of the eggshell surfaces at the end of the 25-day storage period (25.8 ± 2.5°C): (A) Control group; (B) T1 (0% extract); (C) T2 (25% extract); (D) T3 (50% extract); (E) T4 (75% extract); (F) T5 (100% extract). Micrograaphs reveal the transition from the naturally porous structure in the control to a sealed, homogeneous surface in the bioactive coated treatments (T4–T5), supporting the physical barrier effect. Magnification: 1000×; Scale bar = 50 µm.

## References

[1] Puglisi M.J., Fernandez M.L. (2022). The health benefits of egg protein. Nutrients.

[2] Malfatti L.H., Zampar A., Galvão A.C., Robazza W.S., Boiago M.M. (2021). Evaluating and predicting egg quality indicators through principal component analysis and artificial neural networks. LWT.

[3] Luo W., Xue H., Xiong C., Li J., Tu Y., Zhao Y. (2020). Effects of temperature on quality of preserved eggs during storage. Poult. Sci..

[4] Rho T.G., Cho B.K. (2024). Non-destructive evaluation of physicochemical properties for egg freshness: A review. Agriculture.

[5] Biesek J., Wlaźlak S., Brzycka Z., Ragus W., Adamski M. (2024). Impact of storage period on hatching eggs quality, extra-embryonic structures, embryo morphometry, hatchability, and Rosa 1 chick quality. Animal.

[6] Şahansoy H., Caner C., Yüceer M. (2024). The shellac and shellac nanocomposite coatings on enhanced the storage stability of fresh eggs for sustainable packaging. Int. J. Biol. Macromol..

[7] Shi X., Liang Q., Wang E., Jiang C., Zeng L., Chen R., Li J., Xu G., Zheng J. (2023). A method to reduce the occurrence of egg translucency and its effect on bacterial invasion. Foods.

[8] Milkievicz T., Badia V., Souza V.B., Longhi D.A., Galvão A.C., Robazza W.S. (2021). Modeling Salmonella inactivation in chicken meat subjected to isothermal and non-isothermal temperature profiles. Int. J. Food Microbiol..

[9] Cortés-Ramírez G.S., Velasco J.I., Plascencia M.A., Absalón A.E., Cortés-Espinosa D.V. (2024). Development of edible coatings based on different biopolymers to enhance the internal shelf-life quality of table eggs. Coatings.

[10] Jariyapamornkoon N., Phongthajitr C., Sritharet N., Sutthitham W. (2023). Preservation of chicken egg quality using pectin derived from water hyacinth. Appl. Food Res..

[11] Huang J., Hu Z., Hu L., Li G., Yao Q., Hu Y. (2021). Pectin-based active packaging: A critical review on preparation, physical properties and novel application in food preservation. Trends Food Sci. Technol..

[12] Gao M., Wang X., Lin J., Liu X., Qi D., Luo Y., Aheyeli-Kay Y., Ma H. (2023). Separation, structural identification and antibacterial activity of pectin oligosaccharides derived from seed melon. Food Biosci..

[13] Vy Phan T.T., Santhamoorthy M., Thirupathi K., Lin M.C., Kim S.C., Kumarasamy K. (2026). Bioactive antimicrobial polymer membranes: Synthesis, characterization, and their applications in various sectors. J. Mater. Sci..

[14] Medina-Jamarillo C., Ochoa-Yepes O., Bernal C., Famá L. (2017). Active and smart biodegradable packaging based on starch and natural extracts. Carbohydr. Polym..

[15] Bodini R.B., Pugine S.M.P., Melo M.P., Carvalho R.A. (2020). Antioxidant and anti-inflammatory properties of orally disintegrating films based on starch and hydroxypropyl methylcellulose incorporated with *Cordia verbenacea* (erva baleeira) extract. Int. J. Biol. Macromol..

[16] Camargo S.D., Fernandes I.A., Nascimento L.H., Puton B.M.S., Cansian R.L., Steffens C., Zeni J., Paroul N. (2025). Biological activities of essential oil and leaf extracts from Erva Baleeira (*Cordia verbenacea* DC). Food Humanit..

[17] Rodrigues W.D., Cardoso F.N., Baviera A.M., Santos A.G. (2023). In vitro antiglycation potential of erva-baleeira (*Varronia curassavica* Jacq.). Antioxidants.

[18] Oza M.J., Kulkarni Y.A. (2017). Traditional uses, phytochemistry and pharmacology of the medicinal species of the genus *Cordia* (Boraginaceae). J. Pharm. Pharmacol..

[19] Carvalho P.M., Rodrigues R.F.O., Sawaya A.C.H.F., Marques M.O.M., Shimizu M.T. (2004). Chemical composition and antimicrobial activity of the essential oil of *Cordia verbenacea* D.C. J. Ethnopharmacol..

[20] Passos G.F., Fernandes E.S., da Cunha F.M., Ferreira J., Pianowski L.F., Campos M.M., Calixto J.B. (2007). Anti-inflammatory and anti-allergic properties of the essential oil and active compounds of *Cordia verbenacea*. J. Ethnopharmacol..

[21] Belwal T., Cravotto C., Prieto M.A., Venskutonis P.R., Daglia M., Devkota H.P., Baldi A., Ezzat S.M., Gómez-Gómez L., Salama M.M. (2022). Effects of different drying techniques on the quality and bioactive compounds of plant-based products: A critical review on current trends. Dry. Technol..

[22] ABNT, Associação Brasileira de Normas Técnicas (2009). NBR 15679: Ovos Comerciais—Amostragem e Avaliação.

[23] Faiza M., Amina K.T.N., Lynda A., Thoraya D., Ilhem Y.W., Mosadak H.T., Ouardia K., Nassim O. (2022). Evolution of the bacterial contamination rates of egg internal contents during its conservation: First report in Algeria. Veterinaria.

[24] R Core Team (2025). R: A Language and Environment for Statistical Computing.

[25] Lê S., Josse J., Husson F. (2008). FactoMineR: An R package for multivariate analysis. J. Stat. Softw..

[26] Kassambara A., Mundt F. (2020). R Package.

[27] Liaw A., Wiener M. (2002). Classification and regression by randomForest. R News.

[28] Caner C. (2005). The effect of edible eggshell coatings on egg quality and consumer perception. J. Sci. Food Agric..

[29] Alleoni A.C.C., Antunes A.J. (2001). Haugh unit as a measure of the quality of hen eggs stored under unregulated conditions. Sci. Agric..

[30] Ezazi A., Javadi A., Jafarizadeh-Malmiri H., Mirzaei H. (2021). Development of a chitosan-propolis extract edible coating formulation based on physico-chemical attributes of hens’ eggs: Optimization and characteristics edible coating of egg using chitosan and propolis. Food Biosci..

[31] Pires P.G.S., Pires P.D.S., Cardinal K.M., Bavaresco C. (2020). The use of coatings in eggs: A systematic review. Trends Food Sci. Technol..

[32] Pasquali F., Manfreda G., Olivi P., Rocculi P., Sirri F., Meluzzi A. (2012). Modified-atmosphere packaging of hen table eggs: Effects on pathogen and spoilage bacteria. Poult. Sci..

