# Supplementary material for: Citric Pectin–Cordia verbenacea Bioactive Coatings to Preserve Egg Quality Under Non-Refrigerated Conditions Using Machine Learning Approaches
Source: Foods. 2026 Mar 4;15(5):879. doi: 10.3390/foods15050879 (PMC12984527; doi:10.3390/foods15050879)
Supplement: Supplementary file 1 [file foods-15-00879-s001.zip › foods-4157426-supplementary.pdf]

## Supplementary Materials

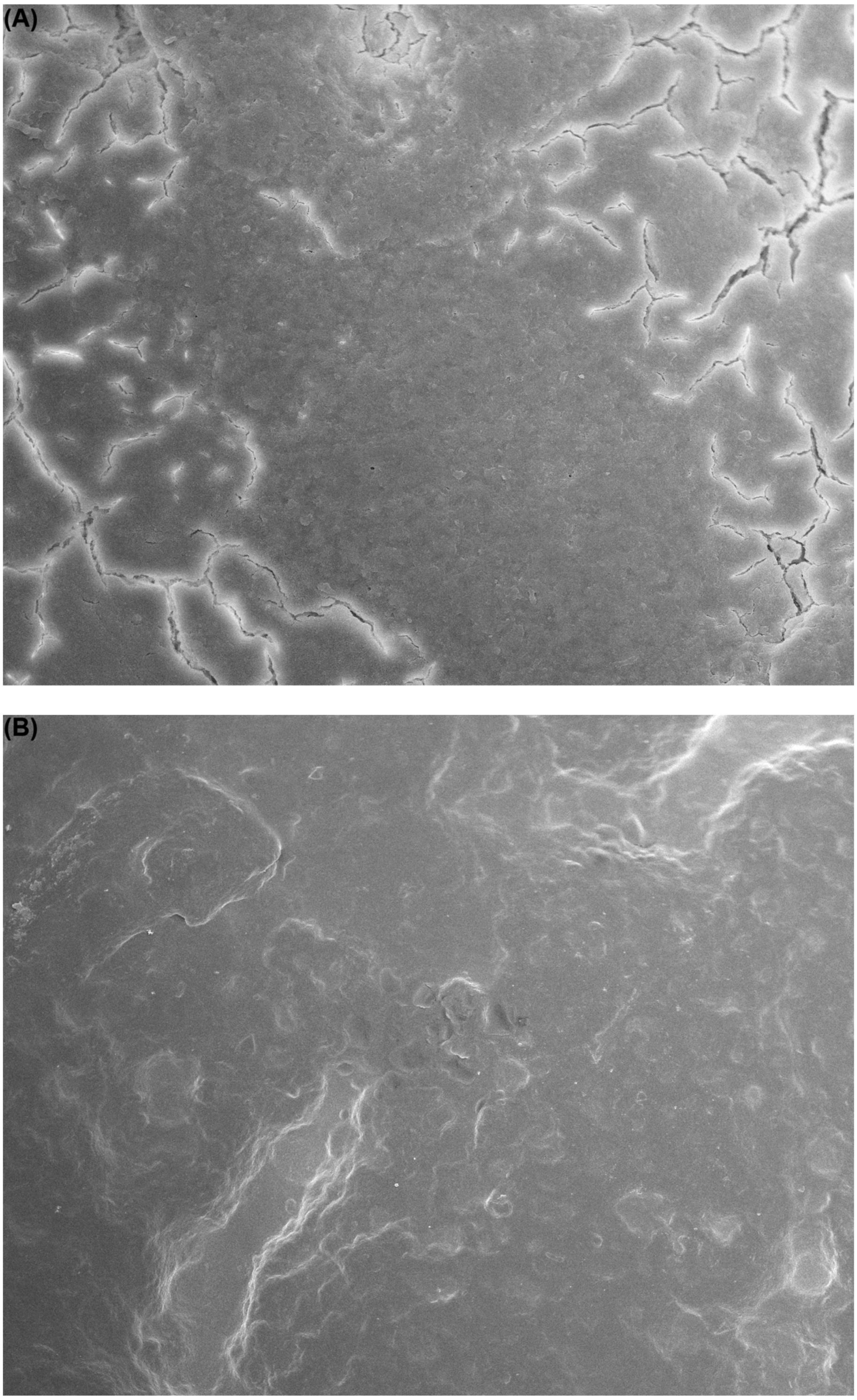

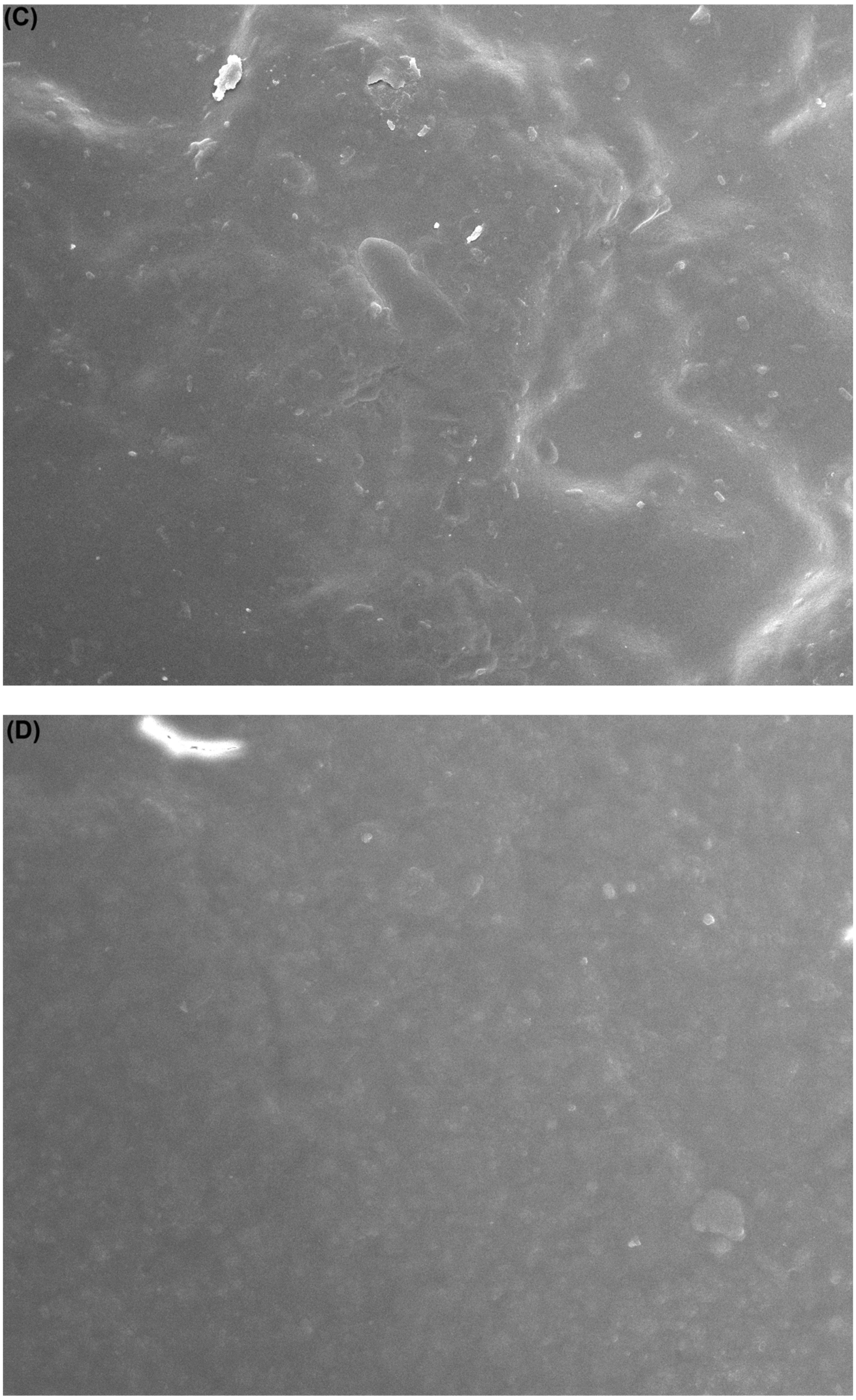
(C)
(D)

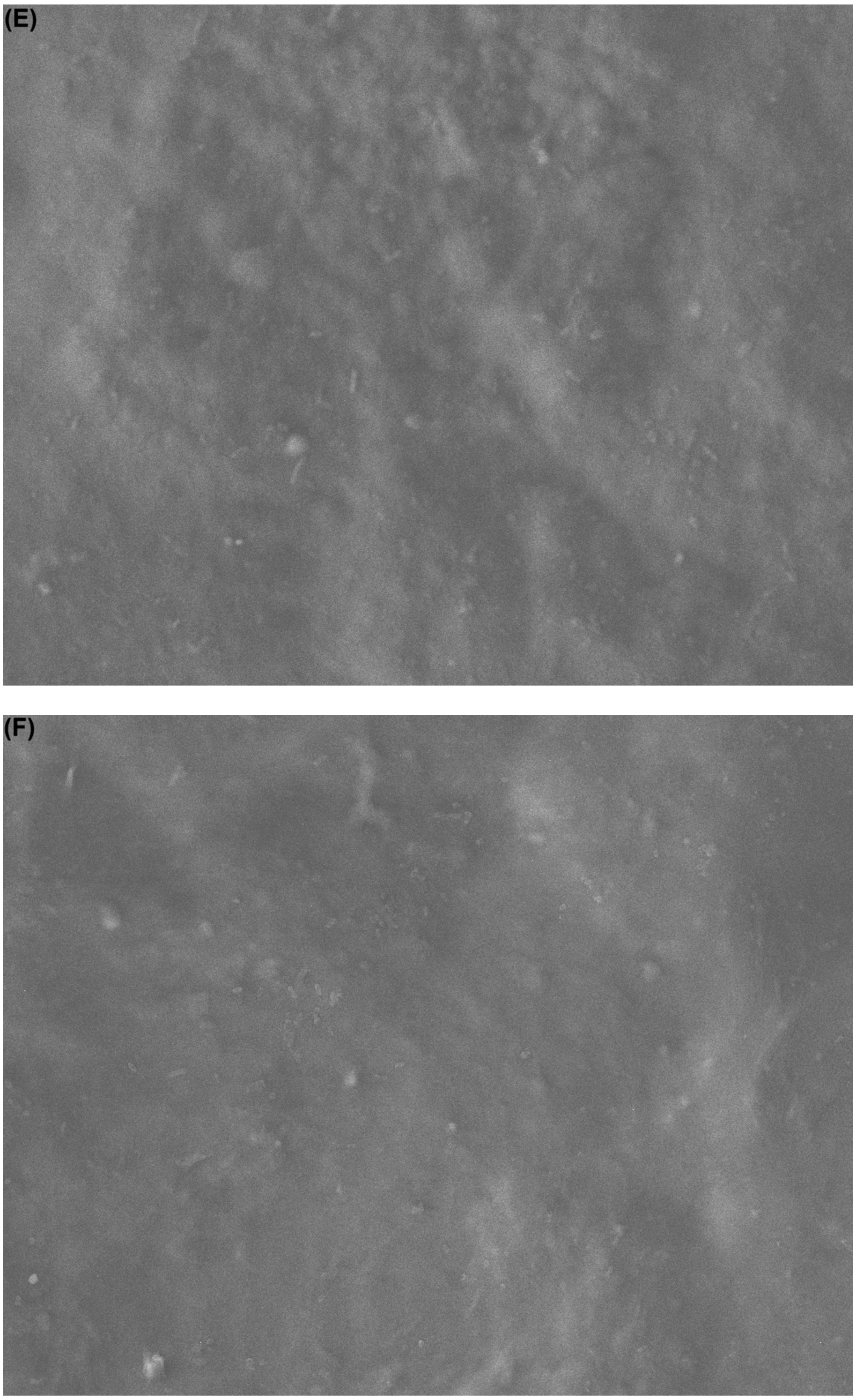


**Figure S1**. Scanning electron microscopy (SEM) micrographs of the eggshell surfaces at the end of the 25-day storage period (25.8 ± 2.5°C): (A) Control group; (B) T1 (0% extract); (C) T2 (25% extract); (D) T3 (50% extract); (E) T4 (75% extract); (F) T5 (100% extract). Micrograaphs reveal the transition from the naturally porous structure in the control to a sealed, homogeneous surface in the bioactive coated treatments (T4-T5), supporting the physical barrier effect. Magnification: 1000x; Scale bar = 50 µm.
